# Pulmonary hypertension-targeted therapies in heart failure: A systematic review and meta-analysis

**DOI:** 10.1371/journal.pone.0204610

**Published:** 2018-10-11

**Authors:** Charles-Antoine Guay, Louis-Vincent Morin-Thibault, Sebastien Bonnet, Yves Lacasse, Caroline Lambert, Jean-Christophe Lega, Steeve Provencher

**Affiliations:** 1 Pulmonary Hypertension Research Group, Laval University, Quebec City, Canada; 2 Institut universitaire de cardiologie et de pneumologie de Québec Research Center, Laval University, Quebec City, Canada; 3 Department of Medicine, Université Laval, Québec, Canada; 4 Hospices Civils de Lyon, Centre Hospitalier Lyon Sud, Service de Médecine Interne-Pathologie Vasculaire, Lyon, France; Freeman Hospital, UNITED KINGDOM

## Abstract

**Background:**

Pulmonary hypertension (PH) due to left heart failure (HF) is the most common form of PH. However, treatment is unclear because there are conflicting results about safety and efficacy of PH-targeted therapies.

**Objectives:**

To assess the effects of PH-targeted therapy on exercise capacity in HF patients.

**Methods:**

MEDLINE, EMBASE and the Cochrane Library were searched from January 1990 to July 2017 for randomized controlled trials comparing PH-targeted therapies to conventional therapy in HF. The primary outcome was to assess the effects on exercise capacity. Secondary outcomes included mortality, hospitalisation, NT-proBNP levels, echocardiographic and hemodynamics parameters and discontinuation rate.

**Results:**

22 studies were included (n = 5448), including 3, 8 and 11 studies with low, high and unknown risk of bias, respectively. PH-targeted therapies were associated with an improvement of exercise capacity (standardized mean difference 0.29;95%CI:0.08–0.50, p = 0.006). Pre-specified subgroup analyses found that this improvement was predominantly observed in studies evaluating phosphodiesterase-5 inhibitors and prostanoids and in patients with reduced ejection fraction. Moreover, systolic pulmonary artery pressure measured by echocardiography was improved (mean difference: -7.5mmHg; [95%CI]: -14.9,-0.1, p = 0.05), which was also entirely driven by studies evaluating phosphodiesterase-5 inhibitors. However, PH-targeted therapies were associated with an increased treatment discontinuation rates and a potential increase in mortality compared to standard treatment.

**Conclusions:**

In conclusion, PH-targeted therapies and especially phosphodiesterase-5 inhibitors may improve exercise capacity in patients with HF. However, an increase in adverse outcomes was likely. Moreover, most studies were at high or unknown risk of bias, precluding confident conclusions about the effects of PH-targeted therapies.

## Introduction

Pulmonary hypertension due to left heart disease (PH-LHD) appears to be the most common form of PH[1]. Epidemiological studies suggest that PH develops in up to 80% of patients with heart failure with preserved (HFpEF) and reduced (HFrEF) ejection fraction [2]. When present, PH-LHD is associated with more severe symptoms and worse exercise tolerance, and exerts a negative impact on outcomes, doubling the risk of mortality as compared to patients with HFpEF/HFrEF without PH[3].

Chronically elevated pressures within the left ventricle (LV) and atrium lead to pathological changes characterized by enlarged and thickened pulmonary veins, pulmonary capillary dilatation, interstitial oedema, alveolar haemorrhage, and lymphatic vessel and lymph node enlargement. In addition to this phenotype of isolated post-capillary PH, the passive elevation in pressures frequently triggers a superimposed precapillary component in some patients, combining pulmonary vasoconstriction, endothelial dysfunction and vascular remodelling[4]. At this stage, pulmonary artery pressure increases further, and this seems to be in excess of the elevation of pulmonary artery occlusion pressure[5]. Although the definition of combined post-capillary and pre-capillary PH is debated[6, 7], recent evidences suggest these patients are at particularly high risk of morbidity and mortality, long-term prognosis being similar to patients with PAH in many series[8].

Importantly, while established treatments may be effective for improving LV function and reducing LV filling pressures, they provide limited improvements in pulmonary vascular remodelling[9]. Some studies suggested that PH-targeted therapies that were shown to improve outcomes in pulmonary arterial hypertension (PAH)[10] could also have a positive effect on endothelial function and the course of PH-LHD[11, 12]. However, most studies were characterized by a small sample size and many had conflicting results. It is thus a matter of concern that drugs approved for the treatment of PAH are commonly used in patients with PH-LHD despite insufficient data supporting their safety and efficacy[13].

We therefore systematically assessed the efficacy and safety of PH-targeted therapies for patients with HFpEF/HFrEF. The aim of the present systematic review and meta-analysis was to assess the effect of PH-targeted therapies for patient with HFpEF/HFrEF on clinically relevant outcomes including exercise capacity, hospitalisation and death compared to conventional therapies.

## Materials and methods

The methods for this systematic review are in accordance with the methodological guidelines for systematic reviews of randomized control trials from «Cochrane Handbook for Systematic Reviews of Interventions»[14]. The complete study protocol is available on PROSPERO (CRD42017083114).

### Study objectives

The primary objective of the study was to determine whether PH-targeted therapies improve exercise capacity compared to conventional therapies alone in HFpEF/HFrEF. Given the heterogeneity in mechanism of action, the primary outcome was assessed according to drug classes. Whenever possible, we also assessed if these outcomes were homogeneous amongst subgroups, including with or without PH (as defined in each study), baseline functional class, HFrEF (<45%) or HFpEF (≥45%), and study duration (<6 months and ≥ 6 months). Secondary objectives were to assess the impact of PH-targeted therapies on mortality, hospitalisation, NT-proBNP, echocardiographic and hemodynamics parameters, as well as treatment discontinuation.

### Search strategy and selection criteria

We searched MEDLINE, EMBASE and the Cochrane Library from January 1990 through July 2017 for randomised controlled trials evaluating PH-targeted therapies for patients with HFpEF/HFrEF. We used free terms and appropriate indexation terms referring to the population and the intervention of interest (*online supplement)*. No filter for randomised controlled trials was used to ensure maximum sensitivity. We also searched for additional articles using the bibliographies of each included studies and any review articles that we retrieved. In addition, we explored grey literature by hand searching the relevant conference abstracts (*online supplement*). Non-English papers were translated to English. Studies were included in the systematic review if they met inclusion criteria defined a priori. Studies had to (1) be prospective randomised controlled trials evaluating the effect of PH-targeted therapies, including prostanoids, endothelin receptor antagonists, type-5 phosphodiesterase inhibitors (PDE5-inhibitors) and soluble guanylate cyclase stimulators compared to conventional therapies in adult with HFpEF/HFrEF of all-causes; (2) have a clear identification of a comparator (placebo or conventional therapies alone); (3) report one of the outcomes of interest of the present systematic review and; (4) have a duration of at least 12 weeks. Titles and abstracts were independently assessed by two reviewers (CAG/LVMT). Relevant papers were then reviewed for a final decision about their inclusion in the review. Reviewers were blinded to authors’ names, journal and year of publication throughout this process. Discrepancies were resolved by consensus or by consulting a third reviewer (SP). Reasons for rejection of citations were kept and the agreement between the two reviewers was measured using the quadratic weighted kappa. Two significant studies were published after the research period and were included in the analysis [15, 16].

### Assessment of methodological quality

The risk of bias of the selected studies was evaluated independently by two reviewers (CAG/LVMT) using the Cochrane’s Risk of Bias Assessment Tool[17]. The reviewers assigned a low, high or unknown risk of bias for each category. A study was considered to have a high risk of bias or an unknown risk of bias if there was at least one category with a high risk or an unknown risk of bias, respectively. Primary analyses were made on all retrieved studies while sensitivity analyses were made including only articles with a low or an unknown risk of bias.

### Data extraction and analysis

The whole process of data extraction was independently made by two reviewers (CAG/LVMT) with tested and validated data collection forms. Retained information included study design, patient characteristics and mean treatment effect on exercise capacity, mortality (all-cause and cardiac-related), hospitalisation (all-cause and cardiac-related), NT-proBNP levels, echocardiographic and hemodynamic parameters and discontinuation rate. 2x2 tables were constructed based on treatment received and available data for the primary and secondary dichotomous outcomes.

Forest plots were created for each outcome. Data were analysed using the Mantel-Haenszel method based on a random-effects model, which accounts for within-study and between-study variability[18]. For continuous outcomes, when multiple scales were used (e.g. exercise capacity), effect sizes were computed using standardized mean difference (SMD) between measures obtained at the end of the study for each comparison group. When the same scale was used, weighted mean difference (MD) were calculated. When mean values or standard deviations were not available, these were estimated as previously described (*online supplement)*[14, 19]. For dichotomous outcomes, risk ratios (RR) were calculated with their 95% confidence intervals (CIs). If one of the cells contained a value of zero, 0.5 was added to each cell, whereas the studies were excluded when there was no event in both groups. When BNP levels where reported, they were transformed in NT-proBNP using a previously published formula[20]. Cochrane’s Q test and I^2^ test were used to assess between study heterogeneity and were considered statistically significant at P<0.10 and I^2^>50%. A sensitivity analysis was also performed using the fixed-effect model.

Subgroup analyses were planned a priori to investigate sources of heterogeneity in the main analyses. Sensitivity analyses were made according to the method used to assess exercise capacity. Sensitivity analysis was also made excluding one trial due to its important weight and its suspiciously delayed publication 15 years after its completion[21]. Publication bias was assessed visually using funnel plots made according to the method used to assess exercise capacity because standardized mean differences are naturally correlated with their standard error and can therefore produce false asymmetry in a funnel plot[14]. All analyses were performed with Review Manager (The Cochrane Collaboration, Oxford, England). The report was written according to the PRISMA statement[22].

## Results

### Characteristics of the selected studies

**Fig 1:**
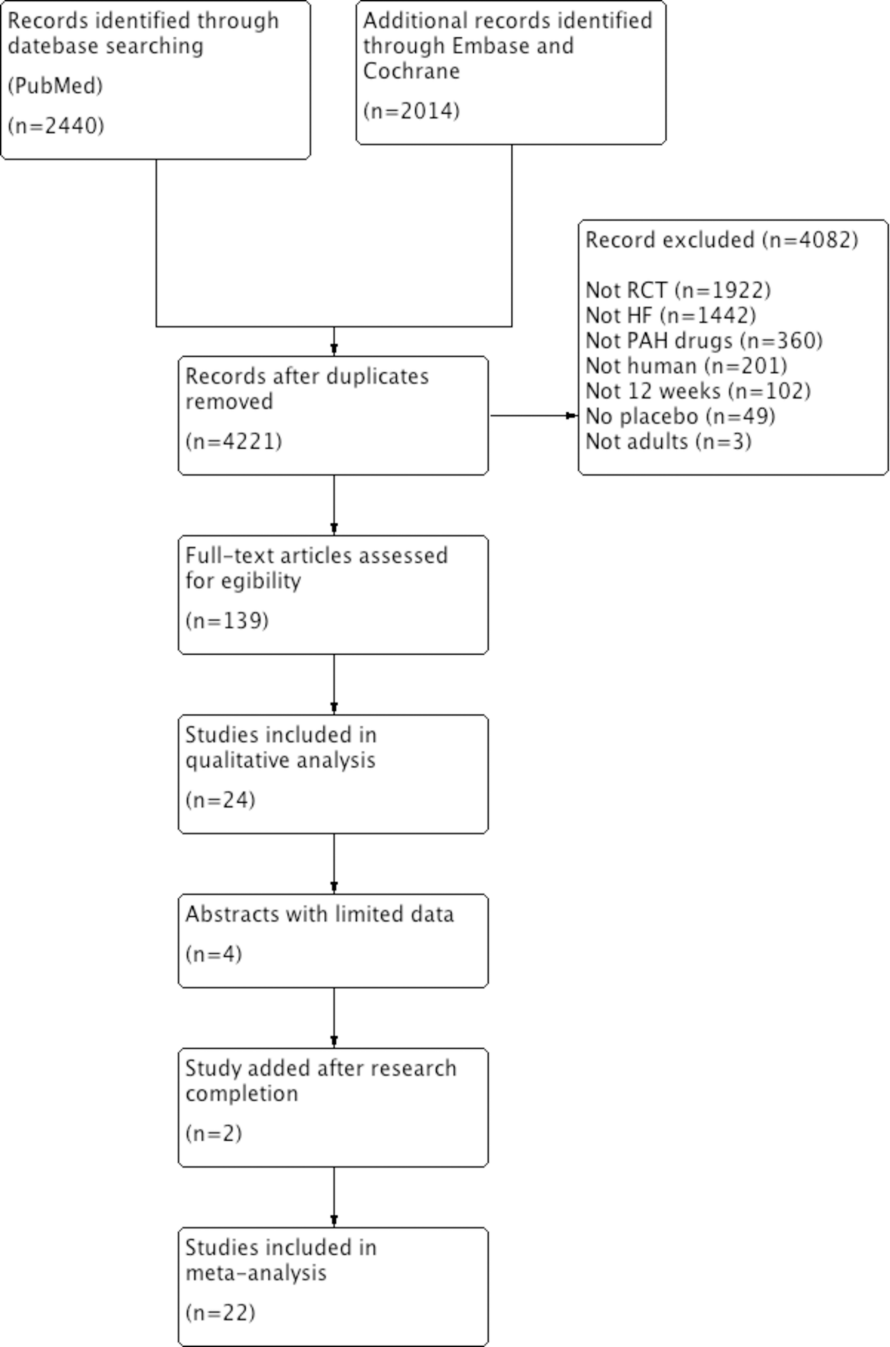
Flow chart.

**Fig 2:**
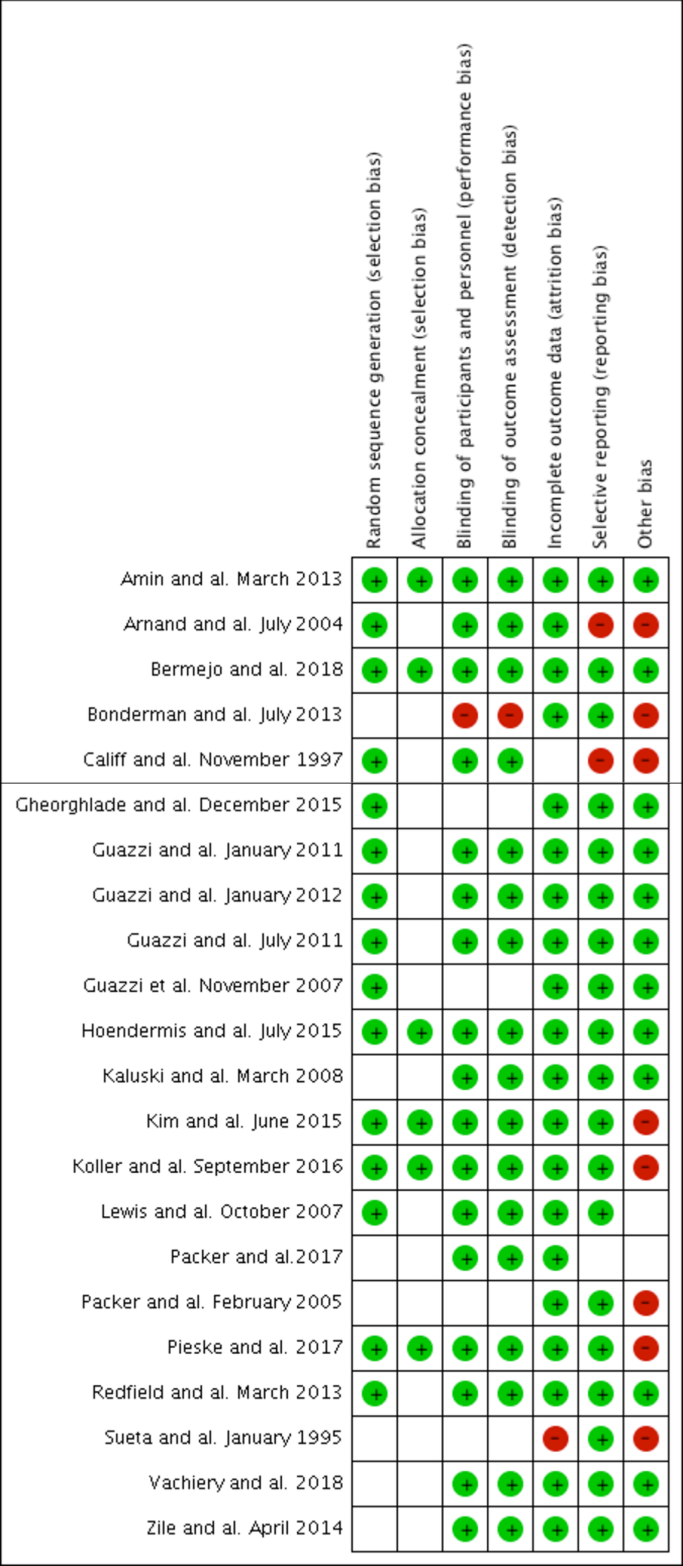
Summary of risk of bias analysis.

Four thousand one hundred and seventy-five studies were retrieved from our literature search. The primary reviewers included 22 [11, 12, 15, 16, 21, 23–39] separate randomized trials representing 5448 patients (quadratic weighted Kappa:0.82;95%CI:0.71–0.94). The reasons for excluding studies appear in **Fig 1.** The included publications are described in **Table 1**. Fifteen studies reported the effects of endothelin receptor antagonists (n = 4)[16, 27, 30, 31], PDE5-inhibitors (n = 8)[11, 12, 23, 25, 26, 32–34], prostanoids (n = 2)[35, 36] and soluble guanylate cyclase stimulators (n = 1)[37] on exercise capacity, whereas 7 other studies [15, 21, 24, 28, 29, 38, 39] were included for the evaluation of secondary endpoints. The duration of the trials ranged from 12 to 52 weeks (median: 22 weeks) and mostly included Caucasian patients with NYHA functional class II or III. Exercise capacity was assessed by 6MWT, VO_2_ max and treadmill in 9, 5 and 1 studies, respectively. Three studies [15, 26, 34] had a low risk of bias, 8 [27, 28, 31, 33, 35–37, 39] had a high risk and 11 [11, 12, 16, 21, 23–25, 29, 30, 32, 38] had an unknown risk (**Fig 2**). Lack of information on allocation concealment was the most frequent reason for unknown risk (**S1 Table**).

**Table 1:** Characteristics of included studies.

Study	n		Length (weeks)	Baseline therapy (%)	Intervention	Female (%)	NYHA (%)	PH	HF type (mean EF±SD)	Outcomes
Phosphodiesterase-5 inhibitors										
Lewis, 2007 [12]	34		12	ACE-1/ARB (83), Diuretics (100), BB (97), MRA (53), Digitalis (68), ICD (86), CRT (29)	Sildenafil 25 to 75 mg TID	15	II (53) III (38) IV (9)	100% P: 33±3 T: 30±2 (mPAP)	HFrEF (<40%) P: 20±2 T: 19±2	**Primary**: VO2 max **Secondary**: 6MWD, hemodynamics, QOL, biomarkers
Guazzi, 2007 [11]	46		26	IECA (80), ARB (17), Furosemide (67), BB (65), MRA (52), Digitalis (26)	Sildenafil 50 mg BID	0	II III	N/A P: 32±3 T: 34±3 (sPAP)	HFrEF (<45%) P: 32±3 T: 31±3	VO2max, brachial artery FMD, ergoreflex, QOL
Guazzi, Jan 2011 [25]	45		52	ACE-I (87), ARB (24), BB (84), MRA (42), Digitalis (11), CRT (38)	Sildenafil 50 mg TID	0	II (42) III (58)	N/A P: 38±3 T: 38±6 (sPAP)	HFrEF (<40%) P: 30±4 T: 30±3	Acute sildenafil response, cardiac dimension, echocardiographic parameters, NT-proBNP, CPET, QOL
Guazzi, July 2011 [24]	44		52	ACE-1/ARB (95), Diuretics (77), BB (82), Digitalis (11)	Sildenafil 50 mg TID	20	N/A	100% P: 36±5 T: 39±5 (mPAP)	HFpEF (>50%) P: 60±6 T: 60±4	Hemodynamics, pulmonary function evaluations, echocardiographic parameters, QOL
Guazzi,2012 [23]	32		52	ACE-I(75), ARB (25), BB (78), MRA (50), Digitalis (9), CRT (53)	Sildenafil 50 mg TID	0	III (91) IV (9)	100% P: 34±3 T: 35±4 (mPAP)	HFrEF (<45%) P: 28±7 T: 29±8	EOB, hemodynamics, VO2 max, QOL
Amin, 2013 [26]	106		12	ACE-I/ARB (94), Diuretics (99), BB (95), MRA (64), Digitalis (27), CRT (7), ICD (9)	Sildenafil 50 mg 3 times a week	26	II (53) III (47)	N/A	HFrEF (<35%)	**Primary**: mean BP and 6MWD **Secondary**: hospitalization, survival
Redfield, 2013 [32]	216		24	ACE-I\ARB (70), Diuretics (86), BB (76), MRA (11)	Sildenafil 60 mg TID	48	II (47) III (53)	N/A P: 43±15 T: 41±14 (sPAP)	HFpEF (>50%) P: 60±7 T: 60±7	**Primary**: changes in VO2max at 24 weeks **Secondary**: composite clinical status score (death, hospitalization, MLHFQ), 6MWD at 12 and 24 weeks, peak sildenafil levels and PCGM at 12 and 24 weeks, left ventricular structure, vascular function, PASP, biomarkers, safety
Kim, 2015 [33]	41		12	ACE-I/ARB (100), Diuretics (100), BB (85), MRA (37), CRT/ICD (7)	Udenafil 100 mg BID	32	II (76) III (24)	N/A P: 43±12 T: 41±9 (sPAP)	HFrEF (<40%) P: 29±7 T: 30±6	**Primary**: change in VO2max at 12 weeks **Secondary**: changes in ventilatory efficiency, LVEF, E/e', LAVI, PASP, NYHA FC at 12 weeks, changes in BNP at 4 and 12 weeks
Hoendermis, 2015 [34]	52		12	ACE-I/ARB (75), Diuretics (90), BB (87), MRA (35)	Sildenafil 60 mg TID	71	II (21) III (79)	100% P: 35±7 T: 35±10 (mPAP)	HFpEF (>45%) P: 58±4 T: 58±4	**Primary**: mPAP **Secondary**: PAWP, CO, VO2max
Bermejo, 2017 [15]	200		24	ACE-I (39), ARB (21), Diuretics (87), BB (48), MRA (42), Digitalis (42), CCB (17)	Sildenafil 40 mg TID	77	I (8) II (48) III (42) (WHO)	100% P: 40±9 T: 38±7 (mPAP)	N/A	**Primary**: composite clinical score (death, HF hospitalisation with diuretics IV, WHO FC, patient’s global assessment) **Secondary**: Adjusted composite score, all-cause mortality, cardiac mortality, HF hospitalisations, changes in 6MWD, WHO FC, BNP, PASP, stroke volume at 6 months
Endothelin receptor antagonist										
Arnand, 2004 [27]		642	24	ACE-I/ARB (93), Diuretics (91), BB (75), MRA (40) Digitalis (46)	Darusentan 10, 25, 50, 100 or 300 mg DIE	18	II (20) III (79) IV (1)	N/A	HFrEF (<35%) P: 27±12 T: 26±11	**Primary**: LVESV **Secondary**: Changes in LVEF, neurohumoral measures, 6MWD, QOL, NYHA class, global assessment, composite clinical status
Packer, 2005 [28]		370	26	ACE-I (89), ARB (12), BB (24), Diuretics (100), MRA (14), Digitalis (74)	Bosentan 500 mg BID	23	III (69) IV (31)	N\A	HFrEF (<35%) P: 23± 7 T: 24±6	**Primary**: clinical status **Secondary**: combined risk of all-cause mortality and worsening HF
Kaluski, 2008 [29]		94	20	ACE-I/ARB (99) Furosemid (99) Thiazide diuretic (23), BB (95) MRA (31)	Bosentan 125 mg BID	29	III (83) IV (17)	100% P: 49±9 T: 52±10 (sPAP)	HFrEF (<35%)	**Primary**: PASP **Secondary**: CI **Exploratory:** others echocardiographic parameters
Zile, 2014 [30]		192	24	ACE-I/ARB (80), Diuretics (77), BB (64)	Sitaxsentan 100 mg DIE	63	II (56) III (44)	N/A	HFpEF (>50%) P: 60±13 T: 61±12	**Primary:** Changes in TET **Secondary**: E/e', LVMI, proportion of subjects achieving improvement, no change or worsening in TET, QOL, NYHA, safety
Koller, 2016 [31]		20	12	ACE-I (45), ARB (40), BB (65) Furosemid (80), Thiazide diuretics (45)	Bosentan 125 mg BID	45	II III	100% P: 66±17 T: 61±17 (sPAP)	HFpEF (>50%) P: 65±7 T: 56±5	**Primary**: 6MWD at 12 weeks **Secondary**: 6MWD at 24 weeks and NT-proBNP, NYHA, echocardiographic parameters of RV, QOL at 12 and 24 weeks
Packer, 2017 [21]		1613	36	ACE-I /ARB (96), BB (51), Loop diuretics (95), MRA (26), Digitalis (58), Nitrates (44), Hydralazine (2), ICD (7)	Bosentan 125 mg BID	26	IIIb (91) IV (9)	N/A	HFrEF (<35%) P: 25±6 T: 25±7	**Primary**: hierarchical clinical composite, all-cause mortality, hospitalization for HF **Secondary**: all cause mortality
Vachiery, 2018 [16]		63	12	ACE-I /ARB (64), BB (68), Loop diuretics (94), MRA (41), Thiazide diuretics (25), CCB (29)	Macitentan 10 mg DIE	65	II (24) III (72)	100% P: 47±11 T: 46±10 (mPAP)	Both N/A	**Primary**: composite of significant fluid retention or worsening in NYHA **Exploratory**: haemodynamic and echocardiographic parameters, NT-proBNP, 6MWD, HF related hospitalisations
Soluble guanylate cyclase stimulators										
Bonderman, 2013 [37]	201		16	ACE-I (71), ARB (28), Furosemide (94), Thiazide diuretics (16), BB (93), MRA (76), CD (60)	Riociguat 0.5,1 or 2 mg TID	14	II (60) III (38) IV (2)	100% P: 40±1 T: 37±2 (mPAP)	HFrEF (<40%) P: 27±5 T: 28±9	**Primary**: Changes mPAP **Secondary**: changes in hemodynamics, echocardiographic parameters **Exploratory**: composite of incidence of clinical worsening, composite of cardiovascular death and hospitalization, QOL, WHO/NYHA class, 6MWD, NT-proBNP
Gheorghlade, 2015 [38]	456		12	ACE-I (61), ARB (23), Diuretics (94), BB (90), MRA (62), ICD (21), CRT-D (7)	Vericiguat 1.25, 2.5, 5 or 10 mg DIE	20	I/II (53) III/IV (47)	N/A	HFrEF (<45%) P: 29±9 T: 30±8	**Primary**: Change in log transformed NT-proBNP **Exploratory**: changes in LVEF, LVEDV, LVESV), clinical events, BP, HR, biomarker levels
Pieske, 2016 [39]	477		12	ACE-I (40), ARB (34), Diuretics (92), BB (80), MRA (37), CCB (36)	Vericiguat 1.25, 2.5, 5 or 10 mg DIE	48	II (55) III/IV (45)	N/A	HFpEF (>45%) P: 57± 7 T: 58±6	**Primary:** Change in log transformed NT-proBNP, change in left atrial volume **Exploratory**: KCCQ, EQ-5D, mortality, morbidity, echocardiography at rest,
Prostanoids										
Sueta, 1995 [36]	33		12	ACE-I (85), Diuretics (100), Digoxin (91)	Max tolerated epoprostenol infusion	12	III (36) IV (64)	N/A	HFrEF (<30%) P: 17±7 T: 17±7	6MWD, LVEF, NYHA, hemodynamics
Califf, 1997 [35]	471		36	ACE-I (84), Diuretics (98), BB (0), Digitalis (91)	Max tolerated epoprostenol infusion	24	III (41) IV (59)	N/A P: 40±9 T: 38±10 (mPAP)	HFrEF (<25%) P: 18±6 T: 17±6	**Primary**: time until death **Secondary**: clinical events, exercise capacity, QOL, resource use

n; numbers of study, NYHA; New York Heart Association, PH; pulmonary hypertension, LVEF; left ventricular ejection fraction, ACE-I; angiotensin convertor enzyme inhibitor, ARB; angiotensin receptor blocker, BB; beta-blocker MRA; mineralocorticoid receptor antagonist; REF; reduced ejection fraction, LVESV; left ventricular end systolic volume, 6MWD; 6 minute walking distance, QOL; quality of life, 6MWT; 6 meters walking test, HF; heart failure, PASP; pulmonary arterial systolic pressure, CI; cardiac index, PEF; preserved ejection fraction, TET; treadmill exercise time, E/e'; early diastolic mitral inflow velocity to early diastolic mitral annular velocity, LVMI; left ventricular mass index, NT-proBNP; N-terminal prohormone of brain natriuretic peptide, RV; right ventricule, ICD; implantable cardioverter defibrillator, CRT; cardiac resynchronisation therapy, P; placebo, T; treatment, VO2max; peak oxygen uptake, FMD; flow mediated dilatation, CPET; cardiopulmonary exercise testing, EOB; exercise oscillatory breathing, BP; blood pressure, MLHFQ; Minnesota Living With Heart Failure Questionnaire, PCGM; plasma cyclic guanosine phosphate, LAVI; left atrial volume, BNP; brain natriuretic peptide, mPAP; mean pulmonary arterial pressure, PAWP; pulmonary arterial wedge pressure, CO; cardiac output, WHO; World Health Organization, LVEDV; left ventricular end-diastolic volume, LVESV; left ventricular end-systolic volume, HR; hearth rate, CCB; calcium channel blocker, KCCQ; Kansas City Cardiomyopathy Questionnaire, EQ-5D; EuroQol-5 dimension.

### Primary outcome

**Fig 3:**
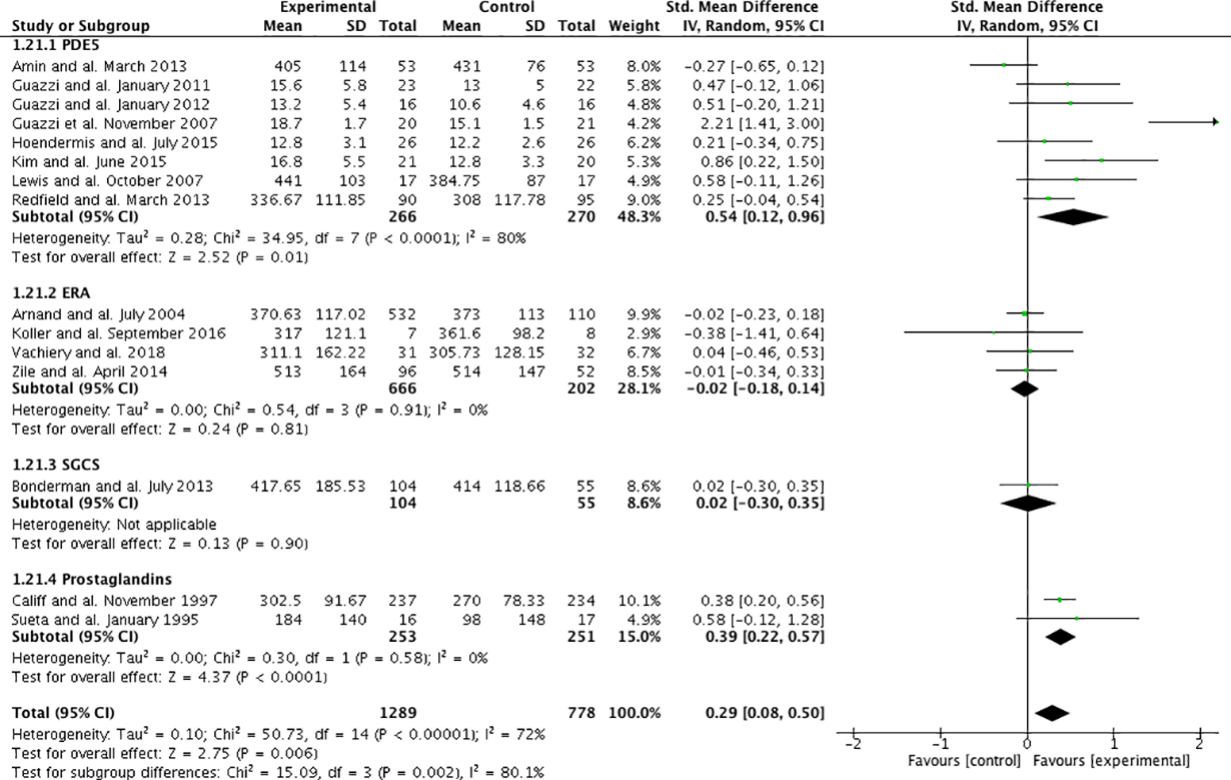
Forest plot of primary outcome.

**Fig 4:**
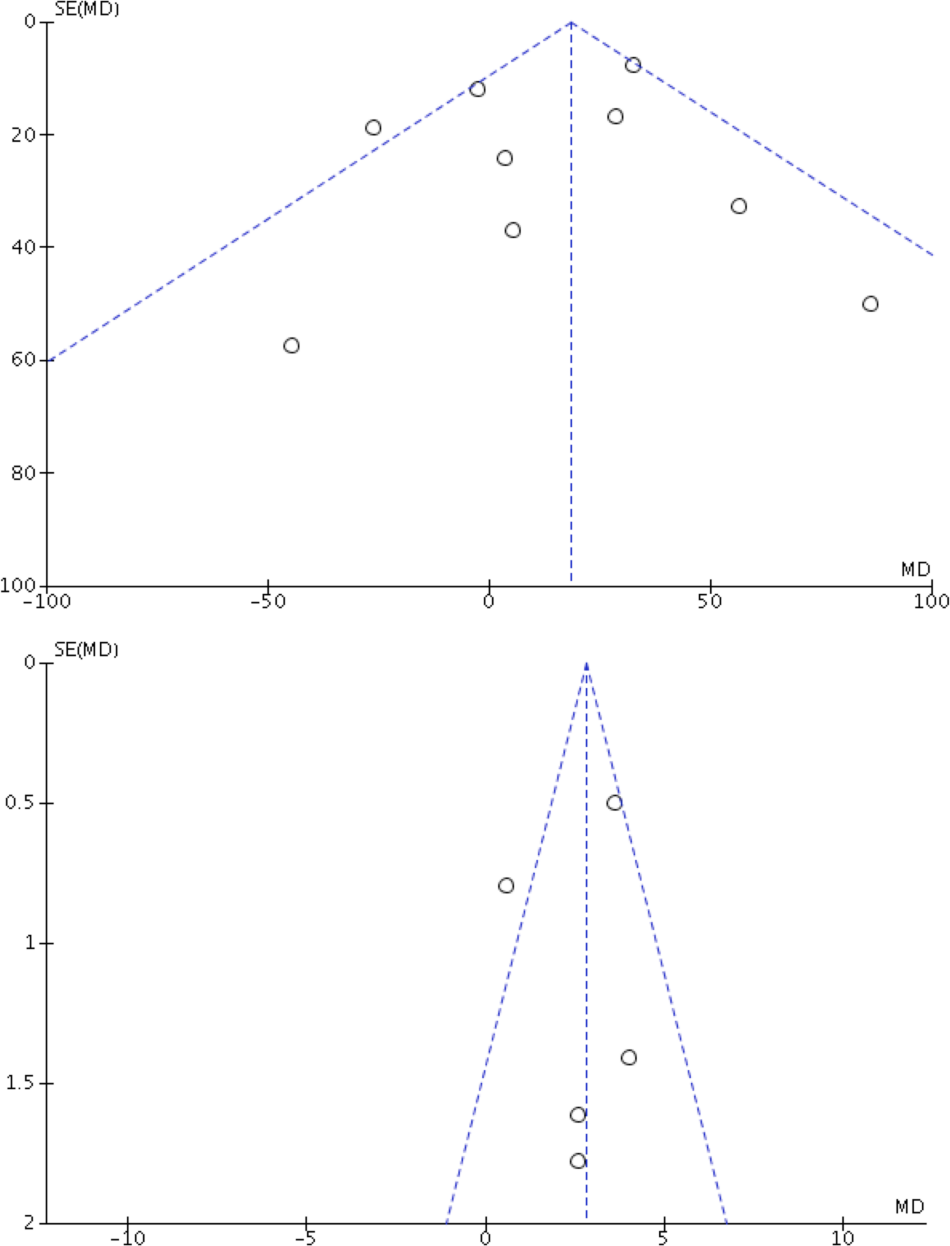
Study of publication bias: funnel plot for the primary outcome of the meta-analysis, including (A) the 6MWT and; (B) VO_2peak_.

PH-targeted therapies were associated with a significant improvement of exercise capacity (SMD:0.29;95%CI:0.08–0.50, p = 0.006) (**Fig 3**). The visual analysis of the funnel plots suggested no publication bias (**Fig 4**). Statistically significant heterogeneity was noted (I^2^ = 72%;p_homogeneity_<0.001). Pre-specified subgroup analyses found that this improvement was observed in studies evaluating PDE5-inhibitors (p_interaction_ = 0.01) and prostanoids (p_interaction_<0.001), as well as studies using VO_2 peak_ as the exercise capacity endpoint (p_interaction_<0.001), studies of longer duration (p_interaction_<0.001) and studies evaluating PH-targeted therapies for HFrEF (p_interaction_ = 0.005) (**Table 2**). Overall, the mean increase in VO_2 peak_ was +2.62ml/kg/min (95%CI; 1.08–4.16, p<0.001), whereas no changes in 6MWD were observed (MD +14; 95%CI:-6-34 meters, p = 0.16). Statistical results from fixed-effect suggested similar confidence intervals results except for changes in 6MWD (MD +18; 95%CI: 8–29 meters, p<0.001). These results were similar when studies with a high risk of bias were excluded (**S1 Fig, S2 Table**), although all studies evaluating prostanoids were at high risk of bias.

**Table 2:** Primary outcome and prespecified subgroups analysis.

Outcomes	All studies								
	n	References	Random effect model		Fixed effects model		Homogeneity		
			SMD	95% CI (p value)	SMD	95% CI (p value)		P value	I^2^ (%)
**Primary outcome**									
Exercise capacity	15	[11, 12, 16, 23, 25–27, 30–37]	0.29	0.08–0.50 (p = 0.006)	0.21	0.11–0.30 (p<0.001)		<0.001	72
**Type of exercise test**									
6MWT	9	[12, 16, 26, 27, 31, 32, 35–37]	MD: 14.1	-5.6–33.7 (p = 0.16)	MD: 18.4	7.8–29.1 (p<0.001)		0.03	53
VO2 max	5	[11, 23, 25, 33, 34]	MD: 2.62	1.08–4.16 (p<0.001)	MD: 2.82	2.1–3.6 (p<0.001)		0.03	64
Treadmill	1	[30]	MD: -1.0	-52.7–50.7 (p = 0.97)	MD: -1.0	-52.7–50.7 (p = 0.97)		N/A	N/A
**Classes of PH-targeted therapies**									
PDE-5i	8	[11, 12, 23, 25, 26, 32–34]	0.54	0.12–0.96 (p = 0.01)	0.33	0.16–0.51 (p<0.001)		<0.001	80
ERA	4	[16, 27, 30, 31]	-0.02	-0.18–0.14 (p = 0.81)	-0.02	-0.18–0.14 (p = 0.81)		0.91	0
sGC stimulators	1	[37]	0.02	-0.30–0.35 (p = 0.90)	0.02	-0.30–0.35 (p = 0.90)		N/A	N/A
Prostanoids	2	[35, 36]	0.39	0.22–0.57 (p<0.001)	0.39	0.22–0.57 (p<0.001)		0.58	0
**Pulmonary hypertension**									
With PH	7	[12, 16, 23, 31, 34, 35, 37]	0.25	0.07–0.42 (p = 0.006)	0.28	0.14–0.41 (p<0.001)		0.30	17
Without PH/Unknown	8	[11, 25–27, 30, 32, 33, 36]	0.40	0.05–0.75 (p = 0.03)	0.15	0.02–0.27 (p = 0.03)		<0.001	83
**Left ventricular ejection fraction**									
Preserved	4	[30–32, 34]	0.13	-0.07–0.33 (p = 0.20)	0.13	-0.07–0.33 (p = 0.20)		0.51	0
Reduced	10	[11, 12, 23, 25–27, 33, 35–37]	0.43	0.13–0.72 (p = 0.005)	0.24	0.23–0.35 (p<0.001)		<0.001	81
Both	1	[16]	0.04	-0.46–0.53 (p = 0.89)	0.04	-0.46–0.53 (p = 0.89)		N/A	N/A
**Study duration**									
≤ 6 months	8	[12, 16, 26, 31, 33, 34, 36, 37]	0.17	-0.10–0.44 (p = 0.21)	0.11	-0.07–0.29 (p = 0.24)		0.05	49
> 6 months	7	[11, 23, 25, 27, 30, 32, 35]	0.40	0.09–0.72 (p = 0.01)	0.24	0.13–0.35 (p<0.001)		<0.001	83
**NYHA functional class**									
Up to II	0	N/A	N/A	N/A	N/A	N/A		N/A	N/A
Up to III	8	[11, 16, 25, 26, 30, 31, 33, 34]	0.36	-0.09–0.81 (p = 0.11)	0.18	0.00–0.36 (p = 0.05)		<0.001	82
Up to IV	7	[12, 23, 27, 32, 35–37]	0.24	0.05–0.42 (p = 0.01)	0.22	0.11–0.33 (p<0.001)		0.05	51

### Secondary outcomes

PH-targeted therapies had no effects on other patients-centered outcomes, except for a significant increase in treatment discontinuation compared to standard treatment (**Table 3**). No differences were noted on safety outcome when comparing studies including only PH patients and studies including patients without PH. However, sensitivity analyses suggested a possible increase in all-cause mortality (RR 1.26; 95%CI:1.04–1.53, p = 0.02, I^2^ = 0% and p_homogeneity_ = 0.70) when the ENABLE study [21] was excluded. Systolic pulmonary artery pressure (sPAP) measured by echocardiography was decreased by PH-targeted drugs and NT-proBNP were not influenced by PH-targeted therapies. However, statistically significant heterogeneity was observed for both measures. Subgroup analyses suggested that decreases in sPAP were driven by studies evaluating PDE5-inhibitors (p_interaction_ = 0.004), whereas decreases in NT-proBNP levels were observed in studies that included patients with HFrEF only (p_interaction_ = 0.02) (**S4 Table**). These results were similar when studies with a high risk of bias were excluded (**S3 and S4 Tables**). Given that PDE5-inhibitors were associated with significant improvements in exercise capacity and sPAP, exploratory analyses were performed and suggested that PDE5-inhibitors were not associated with improvements in patients-centered outcomes. (**S5 Table**).

**Table 3:** Secondary outcomes.

Outcomes	All studies								
	n	References	Proportion of events (%)	Random effect model		Fixed effects model		Homogeneity	
				RR	95% CI (p value)	RR	95% CI (p value)	P value	I^2^ (%)
**Patient centered secondary outcomes**									
All-cause mortality	22	[11, 12, 15, 16, 21, 23–39]	(T) 357/3356 (10.6) (P) 289/2080(13.9) Total: 646/5436(11.9)	1.09	0.92–1.29 (p = 0.32)	1.07	0.93–1.23 (p = 0.34)	0.39	6
Cardiac mortality	20	[11, 12, 15, 16, 23–38]	(T) 20/1022 (2.0) (P) 12/587 (2.0) Total: 32/1609 (2.0)	0.86	0.41–1.80 (p = 0.69)	0.89	0.45–1.75 (p = 0.73)	0.65	0
All-cause hospitalization	10	[11, 12, 15, 23, 25, 27, 28, 31–33, 38]	(T) 180/1362 (13.2) (P) 86/538 (16.0) Total: 266/1900(14.0)	0.85	0.64–1.12 (p = 0.25)	0.85	0.66–1.10 (p = 18)	0.38	7
Cardiac Hospitalization	13	[11, 12, 15, 16, 21, 23, 25, 27, 28, 31, 33, 37, 38]	(T) 366/2289 (16) (P) 242/1407 (17.2) Total: 608/3696(16.5)	0.97	0.74–1.27 (p = 0.81)	1.01	0.87–1.18 (= 0.88)	0.08	41
Treatment discontinuation	22	[11, 12, 15, 16, 21, 23–39]	(T) 544/3120 (17.4) (P) 270/1848 (14.6) Total: 814/4968 (16.4)	1.31	1.15–1.50 (p<0.001)	1.34	1.17–1.54 (p<0.001)	0.84	0
**Hemodynamic outcomes**									
**Outcomes**	**n**	**References**		**Random effect model**		**Fixed effects model**		**Homogeneity**	
				**MD**	**95% CI (p value)**	**MD**	**95% CI (p value)**	**P value**	**I**^**2**^ **(%)**
sPAP	7	[11, 24, 25, 29, 31–33]	N/A	-7.5	-14.9, -0.1 (p = 0.05)	-12.4	-13.5, -11.3 (p<0.001)	<0.001	97
NT-proBNP	10	[12, 25, 27, 31–34, 37–39]	N/A	-240	-578, -98 (p = 0.16)	-214	-324, -104 (p<0.001)	<0.001	78

## Discussion

The present systematic review with meta-analysis documented that PH-targeted therapies may modestly improve exercise capacity in patients with HFpEF/HFrEF. These findings were similar when only studies with a low or an unknown risk of bias were taken into consideration and when the fixed-effects model was used, substantiating the robustness of these results. However, significant heterogeneity was noted and predefined subgroup analyses suggested that this observation was driven by studies 1) of longer duration; 2) evaluating the effects of PDE5-inhibitors or prostanoids; 3) using VO_2 peak_ as the evaluative modality and; 4) recruiting patients with HFrEF. Intriguingly, the presence of PH did not influence the primary outcome. PDE5-inhibitors were also associated with a significant decrease in sPAP and exploratory analyses suggested they might be associated with decreased cardiac-specific hospitalizations in heart failure patient but not in patients with corrected valvulopathy and persistent PH. Importantly, however, these results should be cautiously tempered by the fact that PH-targeted therapies were associated with an increase in treatment discontinuation, that most studies had high or unknown risk of bias and that sensitivity analyses suggested that an increased mortality with PH-targeted therapies cannot be ruled out.

In addition to the passive elevation of blood pressure within the pulmonary circulation, HFpEF/HFrEF has long been recognized to promote venular remodeling, and in some instances, arteriolar remodeling with various combinations of medial hypertrophy, intimal proliferation, adventitial thickening, microthrombi and more rarely fibrinoid necrosis[40]. These histological abnormalities were compatible with clinical and hemodynamic demonstration of a “precapillary component”, either reactive or fixed, of PH-LHD[9]. A hemodynamic definition of these phenotypes has been tentatively proposed, including “isolated post-capillary PH” and “combined post-capillary and pre-capillary PH”, according to a diastolic pressure difference (defined as diastolic PAP minus mean PAWP) <7 mmHg or ≥7 mmHg, respectively[9]. Although the precise characteristics defining this “out-of-proportion PH” and their pathobiological consequences remain controversial [6, 7], the presence of PH-LHD identifies a subgroup of HFpEF [2] and HFrEF [41, 42] at high risk of morbidity and mortality[8]. Interestingly, PH-LHD with a significant precapillary component shares many pathobiological features with PAH. The utilization of pulmonary vasoactive therapies in these patients was thus appealing. As such, numerous randomized controlled trials assessed the effects of these therapies in HFpEF/HFrEF. However, most studies were characterized by a small sample size and many had conflicting results. Some studies even resulted in increased mortality [35] and morbidity events[28].

The heterogeneity in the scales used to assess exercise capacity in included studies led to the use of a SMD for pooled data. SMD can be hard to interpret because the overall intervention effect is not reported with traditional scales. Rules of thumbs have been suggested to interpret effect sizes: 0.2 being a small effect, 0.5 a moderate one and 0.8 a large one[43]. Using this scale, the observed effect size represents a small-to-moderate effect of intervention on exercise capacity. Subgroup analyses suggested that this effect was driven by studies evaluating PDE5-inhibitors and prostanoids. Interestingly, these studies predominantly used the VO_2 peak_ to assess the effects of PH-targeted therapies on exercise capacity, compared to other trials that used the 6MWT. The increase in VO_2 peak_ was similar to the one observed in a previous meta-analysis including shorter trials[44]. The 6MWT and CPET have excellent discriminative properties and have been reported to closely correlate with functional class, disease severity and survival in HFrEF/HFpEF[45]. However, the responsiveness of the 6MWT to detect effective interventions in heart failure studies has been questioned[46]. Therefore, the possibility that other drug classes did not result in improved exercise as a result of the limited evaluative properties of the 6MWT in HFpEF/HFrEF patients cannot be excluded. Our meta-analysis also demonstrated that PH-targeted therapy is associated with a significant decrease in sPAP. This is of clinical interest knowing that elevated sPAP strongly predicts mortality [3], yet a reduction in sPAP is not a surrogate marker for improved survival.

Importantly, PH-targeted therapies were associated with a higher risk of treatment discontinuation. Moreover, sensitivity analyses suggested that an increase in all-cause mortality (RR 1.26; 95%CI:1.04–1.53, p = 0.02, I^2^ = 0% and p_homogeneity_ = 0.70) could not be ruled out with PH-targeted therapies when the ENABLE trial was excluded, despite the fact that this trial was characterized by an increased risk of early worsening in HF necessitating hospitalisation[21]. Consistently, sildenafil increased the risk major clinical events (including hospitalizations) compared to placebo in patients with persistent PH following valvular heart surgery, whereas macitentan almost doubled the incidence of significant fluid retention in PH-LHD in recently published trials[15, 16]. Past observational studies also suggested an increase in morbidity/mortality with the use of other endothelin receptor antagonists [28] or prostanoids [35, 36]. These observations were speculatively explained by cardiac decompensation due to an increasing pulmonary blood flow in the presence of an already elevated left-sided filling pressure and fluid retention[28]. This finding thus supports previous concerns about PH-targeted therapies in HFpEF/HFrEF patients, including PDE5-inhibitors[47]. Conversely, a recent meta-analysis on PDE5-inhibitors in HFrEF/HFpEF observed a reduction of the composite of death or hospitalisation [48, 49], whereas another meta-analysis could not find significant changes in mortality with endothelin receptor antagonists[50]. However, these meta-analyses mainly included trials of short duration. Moreover, when taking into account our results, the significant reduction of this composite outcome observed in PDE5-inhibitors were likely driven by the reduction of hospitalization rather than mortality. Most importantly, recent trials confirmed the frequent disconnection between exercise capacity and the incidence of hard events (e.g. hospitalisations) in PH-LHD [15], underscoring the need of using clinical outcomes (e.g. death or hospitalisation) rather than surrogate outcomes (e.g. exercise capacity or sPAP) in future trials in the field. Taken together, these observation support current recommendations, stipulating that there is currently insufficient evidence supporting the use of PH-targeted therapies in PH-LHD[51].

The present study has several limitations, including the variability in the scale used to assess exercise capacity and in trial duration (ranging from 12 to 52 weeks), the protracted period between the publication of the first and the last trials (around 20 years) and the impossibility to include 4 studies due to unpublished data. The fact that we had to transform median to average and BNP to NT-proBNP using an already published formula is another limitation of our meta-analysis. In addition, most of the studies included in our meta-analysis had methodological flaws with many studies lacking information about key aspects of the methodology, resulting in an unknown or a high risk of bias. Yet, sensitivity analyses excluding studies with high risk of bias resulted in similar improvements suggesting that our results are not explained by the risk of bias. While our subgroup analyses of aggregate data could not find significant differences in exercise capacity, mortality or other clinically important outcomes between these categories of patients, it remains unknown whether meta-analysis of patient-level data would have delineated patterns or specific clinical syndromes predicting treatment responders. Most importantly, the lack of homogeneity in the study population is a major issue. Indeed, the type of HF and the severity of PH were highly variable from one study to the other, and many studies did not even describe whether patients had concomitant PH[11, 21, 25–28, 30, 32, 33, 35, 36, 38, 39]. When assessed, pulmonary hemodynamics were most commonly estimated non-invasively, precluding precise characterization of the PH. These inclusion criteria are somewhat surprising since pulmonary vasoactive therapies could be expected to be mostly effective in patients with a precapillary component of PH. In addition, the prevalence of additional left-sided valvular disease, particularly functional mitral regurgitation, was generally not reported. Finally, whether HF therapy was fully optimized before randomization was generally poorly described. Thus, robust evidence on the safety and efficacy of PH-targeted therapies will require long-term multicentre randomized, controlled trials of hemodynamically phenotyped patients that are clinically stable on optimized background therapy to allow delineating subgroups of patients whom benefit most from PH-targeted therapies[52].

In conclusion, the use of PH-targeted therapies may modestly improve exercise capacity in patients with HFpEF/HFrEF. Subgroup analyses suggested this effect was mostly driven by the use of PDE5-inhibitors. However, PH-targeted therapies were associated with a significant increase in treatment discontinuation and an excess of mortality could not be entirely ruled out. Moreover, most studies were at high or unknown risk of bias and patients’ phenotypic description precluded the delineation a subgroup of patients that could benefit from PH-targeted therapies. Although the potential use of PH-targeted therapies in PH associated with HFpEF/HFrEF is based on a sound pathobiological rationale, these observations do not provide evidence to support the use of these drugs in the clinical management of patients until future trials provide stronger evidence of safety and efficacy.

## Supporting information

S1 Appendix Literature search. (DOCX)

S2 Appendix PRISMA checklist guidance. (DOC)

S1 Fig Forest plot of primary outcome with low and unknown risk of bias studies. (TIF)

S1 Table Detailed evaluation of risk of bias. (DOCX)

S2 Table Primary outcome and prespecified subgroups analysis. (DOCX)

S3 Table Secondary outcomes. (DOCX)

S4 Table Prespecified subgroups analysis for hemodynamics centered secondary outcomes. (DOCX)

S5 Table Exploratory analysis of patients-centered outcomes in trials evaluating PDE5-i. (DOCX)
