# Supplementary material for: Pulmonary hypertension-targeted therapies in heart failure: A systematic review and meta-analysis
Source: PLoS One. 2018 Oct 11;13(10):e0204610. doi: 10.1371/journal.pone.0204610 (PMC6181322; doi:10.1371/journal.pone.0204610)
Supplement: S1 Appendix — (DOCX) [file pone.0204610.s001.docx]

**S1 Appendix: Literature search**

MEDLINE (1990- July 2017), EMBASE (1990- July 2017) and Cochrane Library (1990-July 2017) were searched for randomized placebo-controlled trials evaluating PAH targeted therapies in heart failure. Search terms were designed to provide maximum sensitivity in detecting therapeutic trials in heart failure. Search strategy for ***pubmed*** combined ("heart failure" OR "heart failure"[MeSH Terms] OR "left heart disease") AND (prostaglandin* OR prostanoid* OR prostaglandin i2 OR PGI2 OR prostacyclin OR epoprostenol OR treprostinil OR iloprost OR endothelin receptor antagonist* OR endothelin antagonist*, OR ambrisentan OR bosentan OR macitentan OR phosphodiesterase 5 inhibitor* OR PDE5 inhibitor* OR PDE-5 inhibitor* OR phosphodiesterase type 5 inhibitor* OR sildenafil OR tadalafil OR vardenafil OR solube guanylate cyclase OR riociguat OR prostacyclin receptor agonist OR selexipag OR beraprost) NOT ( child* OR neonat* OR infant* OR newborn*) NOT (animal* OR mouse OR mice OR rat*) AND ( "1990/01/01"[PDat] : "2017/07/06"[PDat]). Search strategy for ***Embase*** were combined ('heart failure'/exp OR 'heart failure' OR 'cardiac failure'/exp OR 'decompensation heart'/exp OR 'left heart disease'/exp) AND ('prostaglandin'/exp OR 'prostanoid'/exp OR 'prostaglandin i2'/exp OR 'pgi2'/exp OR 'prostacyclin'/exp OR 'epoprostenol'/exp OR 'treprostinil'/exp OR 'iloprost'/exp OR 'endothelin receptor antagonist'/exp OR 'endothelin antagonist'/exp OR 'ambrisentan'/exp OR 'bosentan'/exp OR 'macitentan'/exp OR 'phosphodiesterase v inhibitor'/exp OR 'pde5 inhibitor'/exp OR 'pde 5 inhibitor'/exp OR 'phosphodiesterase type 5 inhibitor'/exp OR 'sildenafil'/exp OR 'tadalafil'/exp OR 'vardenafil'/exp OR 'soluble guanylate cyclase'/exp OR 'riociguat'/exp OR 'prostacyclin receptor agonist'/exp OR 'selexipag'/exp OR 'beraprost'/exp) NOT ('child'/exp OR 'neonat' OR 'infant'/exp OR 'newborn'/exp OR 'pediatric'/exp) NOT ('mouse'/exp OR 'mice'/exp OR 'rat'/exp OR 'animal'/exp)) AND [1990-2017]/py. Search strategy for ***Cochrane*** were combined ("heart failure" OR "left heart disease") AND (prostaglandin OR prostanoid OR prostaglandin i2 OR PGI2 OR prostacyclin OR epoprostenol OR treprostinil OR iloprost OR endothelin receptor antagonist OR endothelin antagonist, OR ambrisentan OR bosentan OR macitentan OR phosphodiesterase 5 inhibitor OR PDE5 inhibitor OR PDE-5 inhibitor OR phosphodiesterase type 5 inhibitor OR sildenafil OR tadalafil OR vardenafil OR solube guanylate cyclase OR riociguat OR prostacyclin receptor agonist OR selexipag OR beraprost) NOT ( child OR neonat OR infant OR newborn), in Title, Abstract, Keywords, Publication Year from 1990 to 2017 in Trials.

In addition, we explored grey literature by hand searching the relevant conference abstracts of the American Heart Association, the American College of Cardiology, the European Society of Cardiology, the American Thoracic Society, the American College of Chest Physicians, the European Respiratory Society and the British Thoracic Society published from January 2000 through October 2016.

When mean values or standard deviations were not available, authors were contacted. Otherwise, they were estimated from available data and figures. When data were given as medians, means and standard deviations were obtained using the approach proposed by Wan and colleagues[1]. If only changes from baseline data were given, data at the end of the study were calculated from baseline data. In addition, when standard deviations at the end of the study were not available, they were substituted by the baseline standard deviations[2].
