# Supplementary material for: Pulmonary hypertension-targeted therapies in heart failure: A systematic review and meta-analysis
Source: PLoS One. 2018 Oct 11;13(10):e0204610. doi: 10.1371/journal.pone.0204610 (PMC6181322; doi:10.1371/journal.pone.0204610)
Supplement: S1 Table — (DOCX) [file pone.0204610.s004.docx]

**S1 Table: Detailed evaluation of risk of bias**

| Study | Adequate sequence generation | Allocation concealment | Blinding | Incomplete outcome data | Selective reporting | Other bias | Risk of bias |
| --- | --- | --- | --- | --- | --- | --- | --- |
| **Endothelin receptor antagonists** | | | | | | | |
| Anand, 2004[7] | YES  Patients were randomly allocated with random-number tables | UNCLEAR No information provided | YES Blinding of participants, key personnel and outcome ensured, unlikely that blinding could have been broken | YES Description of withdrawals and method of imputation for missing data | NO Not all outcomes reported | NO  Sponsor Knoll participated in the protocol, manuscript, supplied medication, covered the cost of the study and was responsible for obtaining and analysing data | High |
| Packer, 2005[11] | UNCLEAR Patients randomized, no further information | UNCLEAR No information provided | UNCLEAR Not enough information provided | YES Description of withdrawals and method of imputation for missing data | YES All outcomes are reported | NO  Trial stopped early due to apparent harm | High |
| Kaluski, 2008[19] | UNCLEAR Patients randomized, no further information provided | UNCLEAR No information provided | YES Blinding of participants, key personnel and outcome ensured | YES Description of withdrawals and method of imputation for missing data | YES All outcomes are reported | YES  No other risk of bias | Unknown |
| Zile, 2014[22] | UNCLEAR Patients randomized, no further information | UNCLEAR No information provided | YES Blinding of participants, key personnel and outcome ensured, unlikely that blinding could have been broken | YES Description of withdrawals and method of imputation for missing data | YES All outcomes are reported | YES  No other risk of bias | Unknown |
| Koller, 2016[10] | YES  Randomized by block with the assistance of a drug company | YES Central randomization | YES Blinding of participants, key personnel and outcome ensured, unlikely that blinding could have been broken | YES Description of withdrawals and method of imputation for missing data | YES All outcomes are reported | NO  Pilot study with only a small sample size and was prematurely aborted | High |
| Packer, 2017[23] | UNCLEAR Patients randomized, no further information | UNCLEAR No information provided | YES Blinding of participants, key personnel and outcome ensured | YES Description of withdrawals and method of imputation for missing data | YES All outcomes are reported | UNCLEAR  Possible conflict of interest from the authors sponsored research agreement | Unknown |
| Vachiery, 2018[24] | UNCLEAR Patients randomized, no further information | UNCLEAR No information provided | YES Blinding of participants, key personnel and outcome ensured | YES Description of withdrawals and method of imputation for missing data | YES All outcomes are reported | YES  No other risk of bias | Unknown |
| **PDE5 inhibitors** | | | | | | | |
| Lewis, 2007[20] | YES Randomization was controlled by the hospital pharmacy | UNCLEAR No information provided | YES Blinding of participants, key personnel and outcome ensured, unlikely that blinding could have been broken | YES Description of withdrawals. Missing data balanced in numbers across intervention groups, with similar reasons for missing data across groups | YES All outcomes are reported | UNCLEAR  Possible conflict of interest from the authors sponsored research agreement with Pfizer Inc. | Unknown |
| Guazzi, 2007[15] | YES For participant allocation, a computer-generated list of random number was used | UNCLEAR No information provided | UNCLEAR No information provided | YES Missing outcome date balanced in numbers across interventions groups, with similar reasons for missing data across groups | YES All outcomes are reported | YES  No other risk of bias | Unknown |
| Guazzi, Jan 2011[18] | YES For participant allocation, a computer-generated list of random number was used | UNCLEAR No information provided | YES Blinding of participants, key personnel and outcome ensured, unlikely that blinding could have been broken | YES Descriptions of withdrawals. No information on imputation for missing data. Reasons for missing outcome data unlikely to be related to true outcome | YES All outcomes are reported | YES  No other risk of bias | Unknown |
| Guazzi, Jul 2011[17] | YES For participant allocation, a computer-generated list of random number was used | UNCLEAR No information provided | YES Blinding of participants, key personnel and outcome ensured, unlikely that blinding could have been broken | YES No missing data | YES All outcomes are reported | YES  No other risk of bias | Unknown |
| Guazzi, 2012[16] | YES Randomization was performed on the basis of computer-generated random numbers | UNCLEAR No information provided | YES Blinding of participants, key personnel and outcome ensured, unlikely that blinding could have been broken | YES No missing data | YES All outcomes are reported | YES  No other risk of bias | Unknown |
| Amin, 2013[3] | YES Randomization was controlled by study medication packaging | YES Central randomization | YES Blinding of participants, key personnel and outcome ensured, unlikely that blinding could have been broken | UNCLEAR Description of withdrawals but no information on imputation for missing data | YES Outcomes aren't all described but unlikely to influence study outcome | YES  No other risk of bias | Low |
| Redfield, 2013[21] | YES Patients randomly allocated using a computer random number generator | UNCLEAR No information provided | YES Blinding of participants, key personnel and outcome ensured, unlikely that blinding could have been broken | YES Missing outcome date balanced in numbers across intervention groups with similar reasons for missing data across groups | YES All outcomes are reported | YES  No other risk of bias | Unknown |
| Kim, 2015[9] | YES Patients randomly allocated using a computer random number generator | YES Pharmaceuticals were identical in shape and color and were provided in blinded kits | YES Blinding of participants, key personnel and outcome ensured, unlikely that blinding could have been broken | YES Description of withdrawals and method of imputation for missing data | YES All outcomes are reported | NO  Trial stopped early due to apparent benefit | High |
| Hoendermis, 2015[4] | YES Patients randomly allocated using a computer random number generator | YES Pharmaceuticals were identical in appearance and were supplied to the study site in identical-masked kits | YES Blinding of participants, key personnel and outcome ensured, unlikely that blinding could have been broken | YES Description of withdrawals and method of imputation for missing data | YES All outcomes are reported | YES  No other risk of bias | Low |
| Bemerjo, 2017[5] | YES Patients randomly allocated using a computer random number generator | YES Pharmaceuticals were identical in appearance and were supplied to the study site in identical-masked kits | YES Blinding of participants, key personnel and outcome ensured, unlikely that blinding could have been broken | YES Description of withdrawals and method of imputation for missing data | YES All outcomes are reported | YES  No other risk of bias | Low |
| **Prostanoids** | | | | | | | |
| Sueta, 1995[13] | UNCLEAR Patients randomized, no further information | UNCLEAR No information provided | NO  Blinding of participants, key personnel and outcome not ensured | NO Reason for missing outcome likely to be related to true outcome with imbalance in reasons for missing data | YES All outcomes are reported | NO  Open-label | High |
| Califf, 1997[8] | YES Patients were randomly assigned by phone, baseline characteristics balanced | UNCLEAR No information provided | NO  Blinding of participants, key personnel and outcome not ensured | UNCLEAR  No description of the withdrawals for the major end points of the trial | NO Not all outcomes reported for week 36 | NO  Trial stopped early due to higher mortality in the study group | High |
| **Soluble guanylate cyclase stimulators** | | | | | | | |
| Bonderman, 2013[6] | UNCLEAR Patients randomized, no further information | UNCLEAR No information provided | NO  Only the titration phase is blinded | YES Description of withdrawals and method of imputation for missing data | YES All outcomes are reported | NO  Long-term extension study (week 4 to 16) open-label | High |
| Gheorghiade, 2015[14] | YES Patients were randomized in blocks | UNCLEAR No information provided | UNCLEAR No information provided | YES Description of withdrawals and method of imputation for missing data | YES All outcomes are reported | YES  No other risk of bias | Unknown |
| Pieske, 2017[12] | YES Central randomization | YES Central randomization | YES Blinding of participants, key personnel and outcome ensured, unlikely that blinding could have been broken | YES Description of withdrawals and method of imputation for missing data | YES All outcomes are reported | NO  Major protocol deviation | High |
