# Supplementary material for: Pulmonary hypertension-targeted therapies in heart failure: A systematic review and meta-analysis
Source: PLoS One. 2018 Oct 11;13(10):e0204610. doi: 10.1371/journal.pone.0204610 (PMC6181322; doi:10.1371/journal.pone.0204610)
Supplement: S2 Table — (DOCX) [file pone.0204610.s005.docx]

**S2 Table: Primary outcome and prespecified subgroups analysis**

| **Outcomes** | **Low and unknown risk studies** | | | | | | | | | | | | | |
| --- | --- | --- | --- | --- | --- | --- | --- | --- | --- | --- | --- | --- | --- | --- |
|  | **n** | **References** | | **Random effect model** | | | | **Fixed effect model** | | | | **Homogeneity** | | |
|  |  |  |  | **SMD** | | **95 % CI (p value)** | | **SMD** | | **95 % CI (p value)** | | **P value** | | **I^2^ (%)** |
| **Primary outcome** | | | | | | | | | | | | | | |
| Exercise capacity | 9 | [3, 4, 15, 16, 18, 20-22, 24] | | 0.36 | | 0.02-0.70 (p=0.04) | | 0.21 | | 0.06-0.36 (p=0.007) | | <0.001 | | 77 |
| 6MWT | 4 | [3, 20, 21, 24] | | MD: 12.97 | | -23.27- 49.20 (p=0.48) | | MD: 10.46 | | -11.45-32.38 (p=0.35) | | 0.08 | | 56 |
| VO2 max | 4 | [4, 15, 16, 18] | | MD: 2.34 | | 0.52-4.16 (p=0.01) | | MD: 2.73 | | 1.94-3.51 (p<0.001) | | 0.02 | | 71 |
| Treadmill | 1 | [22] | | MD: -1.00 | | -52.70-50.70 (p=0.97) | | MD:  -1.00 | | -52.70-50.70 (p=0.97) | | N/A | | N/A |
| **Classes PH-targeted therapy** | | | | | | | | | | | | | | |
| PDE-5i | 7 | [3, 4, 15, 16, 18, 20, 21] | 0.50 | | 0.04-0.95 (p=0.03) | | 0.29 | | 0.11-0.47 (p=0.002) | | <0.001 | | 81 | |
| ERA | 2 | [22, 24] | 0.01 | | -0.27-0.29 (p=0.96) | | 0.01 | | -0.27-0.29 (p=0.96) | | 0.89 | | 0 | |
| sGC stimulators | 0 | N/A | N/A | | N/A | | N/A | | N/A | | N/A | | N/A | |
| Prostanoids | 0 | N/A | N/A | | N/A | | N/A | | N/A | | N/A | | N/A | |
| **Pulmonary hypertension** | | | | | | | | | | | | | | |
| With PH | 4 | [4, 16, 20, 24] | 0.27 | | -0.03-0.56 (p=0.08) | | 0.27 | | -0.03-0.56 (p=0.08) | | 0.55 | | 0 | |
| Without PH/Unknown | 5 | [3, 15, 18, 21, 22] | 0.43 | | -0.11-0.98 (p=0.12) | | 0.19 | | 0.01-0.36 (p=0.04) | | <0.001 | | 88 | |
| **Left ventricular ejection fraction** | | | | | | | | | | | | | | |
| Preserved | 3 | [4, 21, 22] | 0.15 | | -0.05-0.35 (p=0.15) | | 0.15 | | -0.05-0.35 (p=0.15) | | 0.52 | | 0 | |
| Reduced | 5 | [3, 15, 16, 18, 20] | 0.66 | | -0.10-1.42 (p=0.09) | | 0.34 | | 0.09-0.60 (p=0.009) | | <0.001 | | 87 | |
| Both | 1 | [24] | 0.04 | | -0.46-0.53 (p=0.89) | | 0.04 | | -0.46-0.53 (p=0.89) | | N/A | | N/A | |
| **Study duration** | | | | | | | | | | | | | | |
| ≤ 6 months | 4 | [3, 4, 20, 24] | 0.06 | | -0.27-0.40 (p=0.70) | | 0.01 | | -0.23-0.26 (p=0.91) | | 0.16 | | 41 | |
| > 6 months | 5 | [15, 16, 18, 21, 22] | 0.60 | | 0.05-1.15 (p=0.03) | | 0.32 | | 0.13-0.52 (p=0.001) | | <0.001 | | 85 | |
| **NYHA functional class** | | | | | | | | | | | | | | |
| Up to II | 0 | N/A | N/A | | N/A | | N/A | | N/A | | N/A | | N/A | |
| Up to III | 6 | [3, 4, 15, 18, 22, 24] | 0.37 | | -0.14-0.88 (p=0.16) | | 0.14 | | -0.05-0.33 (p=0.15) | | <0.001 | | 85 | |
| Up to IV | 3 | [16, 20, 21] | 0.32 | | 0.07-0.57 (p=0.01) | | 0.32 | | 0.07-0.57 (p=0.01) | | 0.60 | | 0 | |
