# Supplementary material for: Pulmonary hypertension-targeted therapies in heart failure: A systematic review and meta-analysis
Source: PLoS One. 2018 Oct 11;13(10):e0204610. doi: 10.1371/journal.pone.0204610 (PMC6181322; doi:10.1371/journal.pone.0204610)
Supplement: S3 Table — (DOCX) [file pone.0204610.s006.docx]

**S3 Table: Secondary outcomes**

| **Outcomes** | **Low and unknown risk studies** | | | | | | | |
| --- | --- | --- | --- | --- | --- | --- | --- | --- |
|  | **n** | **References** | **Random effect model** | | **Fixed effects model** | | **Homogeneity** | |
|  |  |  | **RR** | **95 % CI (p value)** | **RR** | **95 % CI (p value)** | **P value** | **I^2^ (%)** |
| **Patient centered secondary outcomes** | | | | | | | | |
| All-cause mortality | 14 | [3-5, 14-24] | 0.95 | 0.79-1.14 (p=0.58) | 0.96 | 0.80-1.16 (p=0.70) | 0.77 | 0 |
| Cardiac mortality | 11 | [3-5, 14-16, 18-20, 22, 24] | 1.35 | 0.54-3.36 (p=0.52) | 1.42 | 0.59-3.45 (p=0.43) | 0.94 | 0 |
| All-cause hospitalization | 6 | [14-16, 18, 20, 21] | 0.74 | 0.51-1.0 (p=0.12) | 0.71 | 0.49-1.03 (p=0.07) | 0.46 | 0 |
| Cardiac Hospitalization | 8 | [5, 14-16, 18, 20, 23, 24] | 1.02 | 0.65-1.5 (p=0.95) | 1.05 | 0.88-1.25 (p=0.57) | 0.04 | 52 |
| Treatment discontinuation | 14 | [3-5, 14-24] | 1.31 | 1.13-1.53 (p=0.0004) | 1.33 | 1.14-1.54 (p=0.0002) | 0.77 | 0 |
| **Hemodynamic outcomes** | | | | | | | | |
| **Outcomes** | **n** | **References** | **Random effect model** | | **Fixed effects model** | | **Homogeneity** | |
|  |  |  | **MD** | **95 % CI (p value)** | **MD** | **95 % CI (p value)** | **P value** | **I^2^ (%)** |
| sPAP | 5 | [15, 17-19, 21] | -10.0 | -18.4- -1.6 (p=0.02) | -12.7 | -13.9, -11.6 (p<0.001) | <0.001 | 98 |
| NT-proBNP | 5 | [4, 14, 18, 20, 21] | -193 | -616-23 (p=0.37) | -310 | -441, -179 (p<0.001) | <0.001 | 79 |
