# Supplementary material for: Pulmonary hypertension-targeted therapies in heart failure: A systematic review and meta-analysis
Source: PLoS One. 2018 Oct 11;13(10):e0204610. doi: 10.1371/journal.pone.0204610 (PMC6181322; doi:10.1371/journal.pone.0204610)
Supplement: S4 Table — (DOCX) [file pone.0204610.s007.docx]

**S4 Table: Prespecified subgroups analysis for hemodynamics centered secondary outcomes**

| **Outcomes** | **Subgroups** | **All studies** | | | | | | | | **Low and unknown risk studies** | | | | | |
| --- | --- | --- | --- | --- | --- | --- | --- | --- | --- | --- | --- | --- | --- | --- | --- |
|  |  | **n** | **References** | **Random effect model** | | **Fixed effects model** | | **Homogeneity** | | **n** | **References** | **Random effect model** | | **Homogeneity** | |
|  |  |  |  | **MD** | **95 % CI (p value)** | **MD** | **95 % CI (p value)** | **P value** | **I^2^ (%)** |  |  | **MD** | **95 % CI (p value)** | **P value** | **I^2^ (%)** |
| sPAP | Class of PAH therapy | | | | | | | | | | | | | | |
|  | PDE5 | 5 | [9, 15, 17, 18, 21] | -11.6 | -19.5, -3.6 (p=0.004) | -13.1 | -14.2, -11.9 (p=<0.001) | <0.001 | 98 | 4 | [15, 17, 18, 21] | -12.84 | -21.84, -3.83 (p=0.005) | <0.001 | 98 |
|  | ERA | 2 | [10, 19] | 59 | -6.3, 18.0 (p=0.34) | 3.34 | -2.2, 8.9 (p=0.24) | 0.19 | 41 | 1 | [19] | 2.30 | -3.50, 8.10 (p=0.44) | N/A | N/A |
|  | SCGs | 0 | N/A | N/A | N/A | N/A | N/A | N/A | N/A | 0 | N/A | N/A | N/A | N/A | N/A |
|  | Prostanoids | 0 | N/A | N/A | N/A | N/A | N/A | N/A | N/A | 0 | N/A | N/A | N/A | N/A | N/A |
|  | Duration | | | | | | | | | | | | | | |
|  | <6 months | 3 | [9, 10, 19] | 0.70 | -8.2, 9.5 (p=0.88) | -0.95 | -5.1, 3.2 (p=0.65) | 0.04 | 70 | 1 | [19] | 2.30 | -3.50, 8.10 (p=0.44) | N/A | N/A |
|  | >6 months | 4 | [15, 17, 18, 21] | -12.8 | -21.8, -3.8 (p=0.05) | -13.3 | -14.5, -12.2 (p=<0.001) | <0.001 | 98 | 4 | [15, 17, 18, 21] | -12.84 | -21.84, -3.83 (p=0.005) | <0.001 | 98 |
|  | Pulmonary hypertension | | | | | | | | | | | | | | |
|  | With PH | 3 | [10, 17, 19] | -4.2 | -29.7, 21.2 (p=0.74) | -21.5 | -24.0 -19.0 (p<0.001) | <0.001 | 98 | 2 | [17, 19] | -12.76 | -42.06, 16.54 (p=0.39) | <0.001 | 99 |
|  | No PH/  Unknown PH | 4 | [9, 15, 18, 21] | -7.7 | -13.0, -2.4 (p=0.005) | -10.1 | -11.3, -8.8 (p<0.001) | <0.001 | 93 | 3 | [15, 18, 21] | -8.08 | -14.26, -1.90 (p=0.01) | <0.001 | 95 |
|  | LVEF | | | | | | | | | | | | | | |
|  | Reduced | 4 | [9, 15, 18, 19] | -7.6 | -12.6, -2.6 (p=0.003) | -10.6 | -11.9, -9.3  (<0.001) | <0.001 | 91 | 3 | [15, 18, 19] | -7.96 | -13.75, -2.17 (p=0.007) | <0.001 | 93 |
|  | Preserved | 3 | [10, 17, 21] | -5.17 | -28.6, 18.3 (p=0.67) | -17.8 | -20.0, -15.5 (<0.001) | <0.001 | 99 | 2 | [17, 21] | -13.73 | -40.98, 13.51 (p=0.32) | <0.001 | 99 |
|  | NHYA FC | | | | | | | | | | | | | | |
|  | Up to II | 0 | N/A | N/A | N/A | N/A | N/A | N/A | N/A | 0 | N/A | N/A | N/A | N/A | N/A |
|  | Up to III | 4 | [9, 10, 15, 18] | -9.3 | -13.8, -4.9 (p<0.01) | -11.2 | -12.5, -9.9 (p<0.001) | <0.001 | 84 | 2 | [15, 18] | -11.82 | -15.83, -7.80 (p<0.001) | 0.003 | 88 |
|  | Up to IV | 2 | [19, 21] | 0.9 | -2.4, 4.1 (p=0.61) | 0.9 | -2.4, 4.1 (p=0.61) | 0.55 | 0 | 2 | [19, 21] | 0.85 | -2.37, 4.  (p=0.61) | 0.55 | 0 |
|  | Unknown | 1 | [17] | -27.6 | -30.4, -24.8 (p<0.001) | -27.6 | -30.4, -24.8 (p<0.001) | N/A | N/A | 1 | [17] | -27.60 | -30.37, -24.83 (p<0.001) | N/A | N/A |
| NTproBNP | Class of PAH therapy | | | | | | | | | | | | | | |
|  | PDE5 | 5 | [4, 9, 18, 20, 21] | -333 | -808, 141 (p=0.17) | -324 | -455, -193 (p<0.001) | <0.001 | 84 | 4 | [4, 18, 20, 21] | -163 | -610, 284 (p=0.47) | <0.001 | 84 |
|  | ERA | 2 | [7, 10] | 67 | -142, 276 (p=0.53) | 67 | -142, 276 (p=0.53) | 0.47 | 0 | 0 | N/A | N/A | N/A | N/A | N/A |
|  | SCGs | 3 | [6, 12, 14] | -349 | -1573, 875 (p=0.58) | -217 | -952, 518  (p=0.56) | 0.08 | 61 | 1 | [14] | -714 | -2399, 972 (p=0.41) | N/A | N/A |
|  | Prostanoids | 0 | N/A | N/A | N/A | N/A | N/A | N/A | N/A | 0 | N/A | N/A | N/A | N/A | N/A |
|  | Duration | | | | | | | | | | | | | | |
|  | <6 months | 7 | [4, 6, 9, 10, 12, 14, 20] | -453 | -958, 52 (p=0.08) | -466 | -615, -316 (p<0.001) | 0.02 | 62 | 3 | [4, 14, 20] | -304 | -793, 185 (p=0.22) | 0.09 | 59 |
|  | >6 months | 3 | [7, 18, 21] | 81 | -81, 243 (p=0.33) | 81.22 | -81, 243 (p=0.33) | 0.60 | 0 | 2 | [18, 21] | 83 | -188, 354 (p=0.55) | 0.31 | 1 |
|  | Pulmonary hypertension | | | | | | | | | | | | | | |
|  | With PH | 4 | [4, 6, 10, 20] | -385 | -834, 63 (p=0.09) | -466 | -618, -314 (p<0.001) | 0.11 | 51 | 2 | [4, 20] | -255 | -845, 336 (p=0.40) | 0.03 | 79 |
|  | No PH/  Unknown PH | 6 | [7, 9, 12, 14, 18, 21] | -82 | -461, 298 (p=0.67) | 57 | -101, 214 (p=0.48) | 0.03 | 60 | 3 | [14, 18, 21] | 70 | -180, 319 (p=0.58) | 0.39 | 0 |
|  | LVEF | | | | | | | | | | | | | | |
|  | Reduced | 6 | [6, 7, 9, 14, 18, 20] | -553 | -1036, -70 (p=0.02) | -321 | -447, -195 (<0.001) | <0.0001 | 82 | 3 | [14, 18, 20] | -510 | -669, -351 (p<0.001) | 0.97 | 0 |
|  | Preserved | 4 | [4, 10, 12, 21] | 125 | -99, 348 (p=0.28) | 125 | -99, 348 (p=0.28) | 0.66 | 0 | 2 | [4, 21] | 113 | -119, 344 (p=0.34) | 0.97 | 0 |
|  | NHYA FC | | | | | | | | | | | | | | |
|  | Up to II | 0 | N/A | N/A | N/A | N/A | N/A | N/A | N/A | 0 | N/A | N/A | N/A | N/A | N/A |
|  | Up to III | 4 | [4, 9, 10, 18] | -638 | -1588, 313 (p=0.19) | -252 | -687, 181 (p=0.25) | 0.03 | 67 | 2 | [4, 18] | 5 | -474, 485 (p=0.98) | 0.35 | 0 |
|  | Up to IV | 6 | [6, 7, 12, 14, 20, 21] | -146 | -537, 246 (p=0.47) | -212 | -325, -98 (p<0.001) | <0.001 | 84 | 3 | [14, 20, 21] | -251 | -812, 309 (p=0.38) | <0.001 | 88 |
|  | Unknown | 0 | N/A | N/A | N/A | N/A | N/A | N/A | N/A | 0 | N/A | N/A | N/A | N/A | N/A |

Given that PDE5 inhibitors were associated with significant improvements in exercise capacity and sPAP, we assessed whether they were specifically associated with patients-centered outcomes. Although exploratory in nature, these analyses suggested that PDE5-inhibitors might be associated with a decrease in cardiac-specific hospitalizations (RR 0.35; 95%CI: 0.15-0.81, p=0.01, I^2^=0% and p_homogeneity_=0.51) (**Table S5**). However, when data from Bermejo and al. [5] is considered no effect is reported on cardiac-specific hospitalizations (RR 0.57; 95%CI:0.20-1.66, p=0.30, I^2^=63% and p_homogeneity_=0.0.02).
