# Supplementary material for: Pulmonary hypertension-targeted therapies in heart failure: A systematic review and meta-analysis
Source: PLoS One. 2018 Oct 11;13(10):e0204610. doi: 10.1371/journal.pone.0204610 (PMC6181322; doi:10.1371/journal.pone.0204610)
Supplement: S5 Table — (DOCX) [file pone.0204610.s008.docx]

**S5 Table: Exploratory analysis of patients-centered outcome in trials evaluating PDE5-i**

| **Outcomes**^$^ | **Studies evaluating PDE5-i** | | | | | | | | |
| --- | --- | --- | --- | --- | --- | --- | --- | --- | --- |
|  | **n** | **References** | **Proportion of events (%)** | **Random effect model** | | **Fixed effects model** | | **Homogeneity** | |
|  |  |  |  | **RR** | **95 % CI (p value)** | **RR** | **95 % CI (p value)** | **P value** | **I^2^ (%)** |
| All-cause mortality | 10 | [3-5, 9, 15-18, 20, 21] | (T) 7/418 (1.7)  (P) 5/376(1.3)  Total: 12/794(1.5) | 1.23 | 0.37-4.13 (p = 0.74) | 1.26 | 0.44-3.64 (p = 0.66) | 0.45 | 0 |
| Cardiac mortality | 10 | [3-5, 9, 15-18, 20, 21] | (T) 3/305(1.0)  (P) 1/296 (0.3)  Total: 4/601 (0.7) | 2.20 | 0.33-14.76 (p = 0.42) | 2.22 | 0.34-14.71 (p = 0.41) | 0.81 | 0 |
| All-cause hospitalization | 6 | [9, 15, 16, 18, 20, 21] | (T) 21/213 (9.9)  (P) 33/200 (16.5)  Total: 54/413(13.1) | 0.63 | 0.36-1.08 (p = 0.09) | 0.60 | 0.36-1.00 (p = 0.05) | 0.41 | 1 |
| Cardiac Hospitalization | 6 | [5, 9, 15, 16, 18, 20] | (T) 37/204(18.1)  (P) 30/193(15.5)  Total: 67/397(16.9) | 0.57 | 0.20-1.66 (p = 0.30) | 1.16 | 0.74-1.80 (p = 0.51) | 0.02 | 63 |
| Treatment discontinuation | 10 | [3-5, 9, 15-18, 20, 21] | (T) 50/418 (9.9)  (P) 32/398 (7.0)  Total: 52/616 (8.4) | 1.49 | 0.98-2.27 (p = 0.06) | 1.46 | 0.97-2.21 (p = 0.07) | 0.84 | 0 |
